# COVID-19 Vaccination Among Patients Receiving Maintenance Renal Replacement Therapy: Immune Response, Real-World Effectiveness, and Implications for the Future

**DOI:** 10.1093/infdis/jiad162

**Published:** 2023-08-04

**Authors:** Nadine Rouphael, Mary Bausch-Jurken

**Affiliations:** Divison of Infectious Diseases, Emory University School of Medicine, Atlanta, Georgia, USA; Moderna, Inc., Cambridge, Massachusetts, USA

**Keywords:** COVID-19, dialysis, immunocompromised, mRNA vaccine, renal replacement therapy, SARS-CoV-2

## Abstract

Chronic kidney disease affects more than 800 million people worldwide and often progresses to end-stage renal disease, which requires maintenance dialysis. Patients receiving dialysis are at higher risk for severe respiratory infections, including SARS-CoV-2 (the causative agent of COVID-19). In addition, many patients who receive dialysis also receive immunosuppressive treatments for conditions such as systemic vasculitis, systemic lupus erythematosus, or malignancies. Many studies have shown that while mRNA COVID-19 vaccines induce some level of immune response in patients receiving dialysis, the magnitude of response is often lower than that of healthy individuals, and responses rapidly wane. Importantly, the risk of COVID-19–related hospitalization and mortality for patients receiving dialysis is 4- to 8-fold higher compared with the general population. In this article, we summarize recent immunogenicity and real-world outcomes of COVID-19 mRNA vaccination among patients receiving dialysis, with a focus on the 3-dose extended primary series and additional (fourth) doses.

Chronic kidney disease (CKD) affects more than 800 million people worldwide and often progresses to end-stage renal disease (ESRD), which requires maintenance dialysis [1, 2]. Patients receiving dialysis are at higher risk for severe respiratory infections, including SARS-CoV-2 (the causative agent of COVID-19), than the general population. This is because of the impaired, rapidly aging immune system of individuals with ESRD. Chronic low-grade inflammation and the high prevalence of comorbidities, such as diabetes and cardiovascular disease, as well as older age, also contribute to the immune system impairment in this population [3–5]. In addition, many patients requiring dialysis also receive immunosuppressive treatments for conditions such as systemic vasculitis, systemic lupus erythematosus, or malignancies [6].

Preventive measures such as vaccination remain critical to ensure patients receiving dialysis are protected against diseases; however, successful protection through vaccination can be challenging in these individuals, as the impaired immune responses to infections have also been associated with reduced immune responses to vaccines. Accordingly, it has been noted that individuals who were nonresponders to hepatitis B vaccination [7] are also less likely to have adequate immune responses to COVID-19 vaccines [8, 9]. Furthermore, many studies have shown that while mRNA COVID-19 vaccines induce some level of immune response in patients receiving dialysis, the magnitude of responses are often lower than that of healthy individuals and responses rapidly wanes [10, 11]. Importantly, the risk of COVID-19–related hospitalization and mortality for patients receiving dialysis is 4- to 8-fold higher compared with the general population [12–14]. Given that patients receiving dialysis remain at high risk of severe COVID-19, it is essential that the immune responses and real-world effectiveness of COVID-19 vaccines in this population are well characterized to better inform clinical and public health practices. Initial studies examining 2 doses of mRNA vaccines in patients receiving dialysis have been extensively summarized in several systematic literature reviews and meta-analyses [15–17]. In this narrative review, we summarize recent immunogenicity and real-world outcomes of COVID-19 mRNA vaccination among patients receiving dialysis, with a focus on the 3-dose extended primary series and additional (fourth) doses.

## IMMUNOGENICITY STUDIES OF mRNA COVID-19 VACCINATION IN PATIENTS RECEIVING MAINTENANCE DIALYSIS

### Immune Responses to 2 Doses of Vaccine

The phase 2 and 3 clinical studies of mRNA-based COVID-19 vaccines [18, 19] did not include patients receiving maintenance dialysis; however, the immunogenicity of 2 doses of mRNA-based vaccines in this population has been characterized in several smaller observational studies. Overall, based on anti-spike (S) immunoglobulin G (IgG) antibody responses, seroresponse rates in patients receiving dialysis following 2 doses of an mRNA vaccine were generally high (92%–98%; Table 1) [9, 20–27]. However, antibody titers were lower in patients receiving dialysis than in the general population [9, 20, 26]. These findings suggest that early immune responses are generated but are less robust in patients receiving dialysis. In contrast, some studies observed significantly lower seroresponse rates in patients receiving dialysis after 2 doses compared with the general population, including for both SARS-CoV-2 IgG and neutralizing antibodies (82%) [27]. Interestingly, despite an overall response of 94.4% based on SARS-CoV-2–specific IgG, a high proportion of patients receiving dialysis had undetectable neutralizing antibodies titers (49% for vaccine-matched SARS-CoV-2 and 77% for the delta variant) [28]. Additionally, varying seropositivity rates have been reported among patients receiving hemodialysis (84.3%) compared with those receiving peritoneal dialysis (92.4%) [17], as well as between patients who received mRNA-1273 (79.8%–97.4%) and those who received BNT162b2 (59.1%–95.7%) [9, 21–23, 25]. The differences observed in seropositivity among mRNA-1273 and BNT162b2 recipients may be related to differing dosages between these 2 vaccines and/or different study populations or assays. Factors shown to be associated with low antibody titers or lack of seroconversion among patients receiving dialysis include older age, non-Black or non-Native American race, use of immunomodulating medication or anticoagulants, history of transplantation, low serum albumin, and longer length of time on dialysis [9, 22–24, 26, 29, 30]. Cellular immunity is thought to play a key role in disease severity [31]. Lower detectable S-specific T-cell responses (as measured by interferon-ɣ levels) were observed in patients receiving dialysis (46.9%–81.0%) compared with healthy individuals (78.0%–94.6%) after a 2-dose mRNA vaccination regimen [9, 20, 32]. These numbers vary between patients who received hemodialysis (64%) compared with those who received peritoneal dialysis (80%), similar to the differences observed in humoral responses between patients receiving hemodialysis and peritoneal dialysis [33].

**Table 1.** Humoral Immune Responses After 2 Doses of an mRNA COVID-19 Vaccine in Patients Receiving Dialysis

First Author	Population	Age, y	mRNA Vaccine Type	Follow-up Period	Immunogenicity Assessment Method	Immunogenicity Outcomes	Other Results
Van Praet [9]	Hemodialysis, n = 543 Healthy individuals, n = 75	Median 75.0 (IQR, 65.0–82.0)	mRNA-1273, BNT162b2	5 wk after dose 2	SARS-CoV-2 S-specific IgG antibodies	Seropositivity: Hemodialysis, 97.7% mRNA-1273; 92.3% BNT162b2 Healthy individuals, 100% mRNA-1273; 100% BNT162b2 Geometric mean titers: Hemodialysis, 4037 BAU/mL mRNA-1273; 1536 BAU/mL BNT162b2 Healthy individuals, 19 069 BAU/mL mRNA-1273; 8060 BAU/mL BNT162b2	Response rates correlated with immunosuppressive therapy, length of dialysis vintage, levels of serum albumin, lymphocyte count, IgG levels, previous SARS-CoV-2 infection, type of vaccine, and responses to hepatitis B vaccine
Sanders [20]	Hemodialysis, peritoneal dialysis, n = 159 Healthy individuals, n = 191	Peritoneal dialysis mean 59.8 (SD 14.3) Healthy individual mean 58.5 (SD 13.0)	mRNA-1273	28 d after dose 2	SARS-CoV-2 S1-specific IgG antibodies	Seroconversion: Hemodialysis, 99.1% Peritoneal dialysis, 100% Healthy individuals, 100% Median concentrations of antibodies: Hemodialysis/peritoneal dialysis, 1650 BAU/mL Healthy individuals, 3186 BAU/mL	Median concentrations of antibodies for patients on dialysis was lower (*P* = .001) than in healthy individuals
Garcia [21]	Hemodialysis, peritoneal dialysis, N = 2367	18–44 y, n = 199 (8.0%) 45–64 y, n = 803 (34.0%) 65–79 y, n = 985 (42.0%) ≥ 80 y, n = 380 (16.0%)	mRNA-1273, BNT162b2	14–28 and 29–60 d after dose 2	Total SARS-CoV-2–specific RBD Ig (IgG and IgM) antibodies	Seroconversion: 14–28 d, 97.4% mRNA-1273; 95.7% BNT162b2 29–60 d, 98.0% mRNA-1273; 96.0% BNT162b2	…
Dimitrov [22]	Hemodialysis, N = 852	Median 71.0 (IQR, 61.0–80.0)	mRNA-1273, BNT162b2	4 wk after dose 2	SARS-CoV-2 S1-specific IgG antibodies	Level of antibodies (>264 BAU/mL): 79.8% mRNA-1273 59.1% BNT162b2	Lower immune responses were associated with older age, immunosuppressants, anticoagulants, low serum albumin, and BNT162b2 vaccine
Anand [23]	Hemodialysis, N = 610	Not reported	mRNA-1273, BNT162b2	Median 29 d after dose 2	SARS-CoV-2–specific total RBD Ig or RBD IgG antibodies	Percentage with vaccine response: 97.2% mRNA-1273 90.4% BNT162b2	Response rates correlated with race, Hispanic ethnicity, length of dialysis vintage, and levels of serum albumin
Hsu [24]	Hemodialysis, peritoneal dialysis, N = 1528	Mean 64.2 (SD 13.5)	mRNA-1273, BNT162b2	14–74 d after dose 2	SARS-CoV-2 S-specific IgG antibodies	Seropositivity: 96% mRNA-1273 87% BNT162b2	Older age, non-Black and non-Native American race, immunomodulating medication, history of transplantation, type of vaccine, and lower serum albumin were associated with lower likelihood of seroresponse
Anand [25]	Not specified, N = 2563	18–44 y, n = 202.0 (7.9%) 45–64 y, n = 1008 (39.3%) 65–79 y, n = 980 (38.2%) ≥ 80 y, n = 373 (14.6%)	mRNA-1273, BNT162b2	14–180 d after dose 2	SARS-CoV-2–specific total RBD Ig or RBD IgG antibodies	Seropositivity: Days 14–30, 97.7% mRNA-1273; 94.7% BNT162b2 Days 121–150, 91.4% mRNA-1273; 78.5% BNT162b2	Seropositivity after mRNA vaccines rapidly waned; Low antibody levels were associated with risk for breakthrough infection
Grupper [26]	Hemodialysis, n = 56 Control (healthcare workers without kidney failure), n = 95	Hemodialysis, mean 74.0 (SD 11.0) Control, mean 57.0 (SD 9.0)	BNT162b2	Median 30 d after dose 2	SARS-CoV-2 RBD-specific IgG antibodies	Positive antibody response: Hemodialysis, 96% Control, 100% Median concentrations of antibodies: Hemodialysis, 2900 AU/mL Control, 7401 AU/mL	Factors associated with lower antibody levels included older age and lower lymphocyte count
Speer [27]	Hemodialysis, n = 22 Healthy controls, n = 46	Hemodialysis, median 74.0 (range, 51.0–92.0) Healthy controls, median 48.0 (range, 28.0–90.0)	BNT162b2	20 d after dose 2	SARS-CoV-2 S1-specific IgG antibodies	Positive response: Hemodialysis, 82% Controls, 100% Median antibody index: Hemodialysis, 6 Controls, 74	…

Abbreviations: BAU, binding antibody unit; Ig, immunoglobulin; IQR, interquartile range; RBD, receptor binding domain; S, spike glycoprotein; S1, subunit 1 of spike glycoprotein.

The durability of humoral responses among patients receiving dialysis after 2 doses of an mRNA vaccine has also been examined in several studies [10, 11, 25]. In a large longitudinal study of 970 patients on dialysis and 125 healthy participants, waning of humoral response for patients receiving dialysis was more pronounced compared with the healthy population, with a respective 68% and 98% of participants maintaining detectable S-specific and receptor binding domain (RBD)-specific IgG antibodies 6 months following vaccination [11]. The decline of antibody titers between 2 and 6 months following vaccination was also faster in patients receiving dialysis (median RBD titers at 2 months, 98.1; at 6 months, 57.7) than healthy individuals (median RBD titers at 2 months, 99.3; at 6 months, 89.0) [11]. In a European study, the factors associated with the faster immunity decline were type of vaccine administered, use of immunosuppressive drugs, male sex, type of dialysis, and length of time on dialysis [11]. Overall, among patients on maintenance dialysis, the magnitude of antibody and T-cell immune responses to 2 doses of mRNA COVID-19 vaccination is lower and immunity wanes faster than in healthy individuals.

### Immune Responses to Additional Doses of mRNA COVID-19 Vaccines

A key approach to mitigating the low immune responses and rapid decline after 2 doses of COVID-19 mRNA vaccines has been the administration of additional doses for high-risk populations, including those with CKD and receiving dialysis [34–36]. The Advisory Committee on Immunization Practices in the United States now recommends that immunocompromised populations receive a 3-dose primary schedule of mRNA-based vaccines (100-µg dose for mRNA-1273 or 30-µg dose for BNT162b2 [≥12 years of age]) to further boost immune responses [34]. In addition, mix-and-match schedules, wherein a heterologous third dose of an mRNA-based vaccine is administered, may also be used for those who received prior vaccination with a non–mRNA-based vaccine [34].

Compared with responses following dose 2, results from studies characterizing the humoral immune responses to a third dose of mRNA vaccines have shown that this regimen increases SARS-CoV-2 S-specific IgG antibody responses among patients receiving dialysis (Table 2) [3, 29, 37–44]. Similar findings were observed among studies evaluating neutralizing antibody responses after 3 doses. Importantly, a study demonstrated that 75% of patients on dialysis with weak or absent (<150 U/mL) antibody responses after 2 mRNA vaccine doses had high (≥150 U/mL) antibody responses to the third dose [45]. Furthermore, a third dose of mRNA vaccine induced a 58.6-fold increase in binding antibody titers and an 18-fold increase in neutralizing antibody titers compared with 2 doses [3, 38].

**Table 2.** Humoral Responses to 3 and 4 Doses of mRNA COVID-19 Vaccines in Patients Receiving Dialysis

First Author	Population	Age, y	Vaccination Regimen	Time Between Doses 2 and 3	Follow-up Period	Immunogenicity Method	Immunogenicity Outcome	Other Results
After Third Dose								
Kohmer [37]	Hemodialysis, peritoneal dialysis, N = 194	Mean 69.6 (SD 14.2)	2 doses of BNT162b2 and a third dose of mRNA-1273	<6 mo	4 wk after dose 2 4 wk after dose 3	SARS-CoV-2 RBD-specific IgG antibodies	Seroconversion: 2 doses, 56.6% 3 doses, 100% Mean antibody levels: 2 doses, 1192 BAU/mL 3 doses, 23 120 BAU/mL	Third dose increased antibody titers in individuals who had weak seroresponse after 2 doses
Clavero [29]	Hemodialysis, N = 208	Mean 62.6 (SD 15.6)	2 doses of BNT162b2 or CoronaVac, then a third dose of BNT162b2	8 mo	2 and 4 mo after dose 2 4 mo after dose 3	SARS-CoV-2 S1-specific IgG antibodies	Seroresponse: 2 doses BNT162b2, 2 mo follow-up, 65.4% BNT162b2, 4 mo follow-up, 88.9% 3 doses BNT1262b2, 98.8%	Predictors of responsiveness to third dose included age and vaccine type
Beilhack [38]	Peritoneal dialysis, N = 27	Mean 54.3 (range, 33.0–76.0)	3 doses of mRNA-1273	7 mo	6 mo after 2 doses 1 mo after dose 3	SARS-CoV-2 RBD-specific IgG antibodies	Antibody titers: Third dose induced a 58.6-fold increase in antibody levels relative to after dose 2	…
Patyna [39]	Hemodialysis, N = 42	Median 62.0 (IQR, 52.0–72.5)	2 doses of ChAdOx1 or BNT162b2, then a third dose of mRNA-1273	6 mo	4 wk after 2 doses 14 d after third dose	SARS-CoV-2 RBD-specific IgG antibodies SARS-CoV-2 neutralizing activity	Percentages with IgG antibodies: 2 doses, 88.1% 3 doses, 89.2% Neutralizing activity: 2 doses, 76.2% 3 doses, 94.6%	Third dose significantly increased IgG antibody levels and neutralizing activity relative to dose 2
Wang [40]	Hemodialysis (no prior infection), N = 42	Mean 63 (SD 10)	3 doses of mRNA-1273	Not specified	Not applicable	SARS-CoV-2 surrogate neutralizing antibodies (wild-type or omicron)	50% neutralization titers: After dose 3, titers against wild-type and omicron increased 18- and 23-fold, respectively, relative to after dose 2	…
Quiroga [41]	Hemodialysis, N = 711	Median 67 (range, 20–89)	2 doses of mRNA-1273, BNT162b2, ChAdOx1, or Ad26.COV2.S, then a third dose of mRNA-1273 or BNT162b2	<6 mo, 77% of patients > 6 mo, 23% of patients	6 and 9 mo after dose 2	SARS-CoV-2 S-specific IgG antibodies	Seroconversion: 9% of participants had no detectable antibodies at 6 mo after dose 2; among these patients, 91% seroconverted after a third dose	…
Garcia [42]	Not specified, 2 doses, n = 3041; 3 doses, n = 2720	2 doses: 18–44 y, n = 225 45–64 y, n = 892 65–80 y, n = 1316 ≥ 80 y, n = 608 3 doses: 18–44 y, n = 229 45–64 y, n = 1026 65–80 y, n = 1032 ≥ 80 y, n = 433	2 doses of mRNA-1273, BNT162b2, or 1 dose of Ad26.COV2.S, then an additional (second or third) dose of mRNA-1273 or BNT162b2	Median 189 d	14–60 d after dose 2 or dose 3	SARS-CoV-2 RBD-specific IgG antibodies (stratified by IgG index values of <10, 10–23, or >23)	RBD IgG index value >23: 2 doses, 81% 3 doses, 97%	Among patients who had RBD IgG index value of <10 after 2 doses (associated with higher risk of breakthrough infection), 82% had an index level >23 after dose 3
After Fourth Dose								
Becker [43]	Hemodialysis, N = 50	Median 69.5 (IQR, 60.0–79.0)	3 doses of BNT162b2, then a fourth dose of mRNA-1273	Time between doses 2 and 3, 6 mo Time between doses 3 and 4, 4 mo	28 d and 4 mo after dose 3 21 d after dose 4	SARS-CoV-2 surrogate neutralizing activity (original [B.1], delta, and omicron [BA.1] strains)	Percentage of samples >20% inhibition threshold: Original strain, 3 doses 64%; 4 doses 96% delta, 3 doses 64%; 4 doses 94% omicron, 3 doses 38%; 4 doses 80%	Similar results were found for SARS-CoV-2 S-specific IgG antibody responses
Affeldt [44]	Hemodialysis, 3 doses, n = 309; 4 doses, n = 182	3 doses: median 68.0 (IQR, 23.8) 4 doses: median 66.0 (IQR, 23.5)	4 doses of mRNA-1273 or BNT162b2	Median time between doses 2 and 3, 188 d Median time between doses 2 and 4, 308 d	40 d after third dose 34 d after fourth dose	SARS-CoV-2 serum surrogate neutralizing activity (wild-type or omicron BA.1)	Samples above neutralizing antibody cutoff: Wild-type, 3 doses 88%; 4 doses 95% omicron BA.1, 3 doses 31%; 4 doses 64%	…

Abbreviations: BAU, binding antibody unit; IgG, immunoglobulin G; IQR, interquartile range; RBD, receptor binding domain; S, spike glycoprotein; S1, subunit 1 of spike glycoprotein.

Although administration of a third vaccine dose can improve antibody responses in patients receiving dialysis similar to vaccinated healthy individuals, the durability of responses appears to be limited. In a longitudinal study of patients receiving dialysis, neutralization activity against SARS-CoV-2 declined 3-fold during 121 days of follow-up after a third mRNA vaccine dose; however, subsequent administration of a fourth dose increased antibody titers [43]. Moreover, a fourth dose has been shown to further seroconvert those who failed to do so after 3 doses [46]. Importantly, a fourth dose of an mRNA vaccine also enhanced neutralizing activity against the omicron variants of concern (from 30%–38% after dose 3 to 64%–80% of participants after dose 4) [43, 44], although responses were lower than those against the ancestral strain. Accordingly, updated recommendations in countries such as the United States include a fourth dose of an mRNA vaccine, and more recently, recommendations were further updated to include updated vaccines that include mRNAs encoding for the ancestral SARS-CoV-2 and an omicron variant of concern to broaden protection against SARS-CoV-2 variants [47]. Indeed, in a preliminary study where bivalent vaccination was administered as a fifth dose to patients receiving dialysis, a significant increase in anti-S IgG and neutralization activity was observed against the BA.4 and BA.5 omicron subvariants [48]. However, additional evidence is currently limited for these newer variant-updated vaccines among patients receiving dialysis.

Unlike rapid changes in humoral immunity, cellular responses to a third or fourth vaccine dose were more stable over time; the frequency of activated T cells increased slightly after a third or fourth dose and declined moderately over time [43]. Taken together, the administration of additional doses of mRNA vaccine can enhance immune response against SARS-CoV-2 in patients receiving maintenance dialysis.

## REAL-WORLD OUTCOMES OF COVID-19 VACCINATION IN PATIENTS RECEIVING DIALYSIS

The authorization of mRNA COVID-19 vaccines and their subsequent worldwide distribution has allowed for rapid accumulation of evidence on real-world outcomes of mRNA vaccines among patients receiving dialysis. In the largest study in the United States to date examining the effectiveness of mRNA COVID-19 vaccination in patients receiving dialysis (January 1 to February 25, 2021), vaccination with a single dose of BNT162b2 (n = 12 169) or mRNA-1273 (n = 23 037) reduced the risk of disease compared with no vaccination (78% and 73% lower relative risk, respectively), COVID-19–related hospitalization (adjusted odds ratio [aOR], 0.50; 95% confidence interval [CI], .35–.70 and aOR, 0.68; 95% CI, .51–.91, respectively), and death (aOR, 0.29; 95% CI, .13–.58 and aOR, 0.35; 95% CI, .19–.58, respectively) [49]. Similarly, results from another large retrospective study of 16 213 patients receiving dialysis in the United States (February 2021 to December 2021) indicated that the effectiveness of 2 doses of mRNA vaccine was 45% (mRNA-1273, 50%; BNT162b2, 37%) against COVID-19 infection and 53% against hospitalization or death (mRNA-1273, 63%; BNT162b2, 39%) [50]. However, it remains notable that vaccine effectiveness declined during a later period when the delta variant was the predominant strain. In support of these 2 large studies, smaller studies in the United Kingdom (December 2020 to September 2021), Italy (November 2021 to February 2022), and Japan (December 2021 to May 2022) reported similar findings: mRNA vaccination (2–3 doses) improved COVID-19 outcomes for patients requiring dialysis [51–53]. Interestingly, a meta-analysis study of 7 studies reported a 47% decrease in the risk of COVID-19–related hospital admissions for patients who received 3 doses of mRNA vaccine compared with 2 doses, but no significant differences in the risk of breakthrough infections or all-cause death [54]. Of note, there are currently no published reports on the effectiveness of the bivalent COVID-19 vaccines or the doses beyond the extended 3-dose primary series in patients receiving dialysis. There are also no reports on the effectiveness of the vaccines after January 2022 when the omicron variants became predominant in the population of patients requiring dialysis. In summary, mRNA vaccination improves real-world outcomes stemming from SARS-CoV-2 infection, such as severe disease and death, in patients receiving maintenance dialysis.

## CONCLUDING REMARKS

Patients receiving maintenance dialysis remain at higher risk of SARS-CoV-2 infection and severe COVID-19 than healthy individuals [14]. More steps need to be taken to sufficiently protect this population from disease. While optimal COVID-19 vaccination regimens were not investigated for these patients in large clinical trials, a number of smaller prospective and observational studies have been conducted to characterize the immunogenicity elicited by these vaccines in patients receiving dialysis. In this review, we summarized the recent key studies of mRNA COVID-19 vaccines in patients receiving dialysis. Overall, a 2-dose mRNA vaccination regimen induces humoral and cellular immune responses that are lower than those in healthy individuals, with more rapidly waning antibody responses. However, with the subsequent administration of third and fourth doses, antibody responses can be increased. Furthermore, despite lower humoral responses, 2 or more doses of an mRNA-based vaccine can significantly reduce the odds of severe COVID-19, hospitalization, and death in patients receiving dialysis.

Protecting vulnerable populations, including those receiving dialysis, has been a primary focus of public health policies throughout the COVID-19 pandemic, with current guidelines in the United States recommending 3 or more doses of mRNA vaccines as the most effective way of preventing serious disease [34]. However, as the COVID-19 pandemic continues to evolve, continued efforts to complement vaccination strategies with early detection and treatments in these populations is essential. Assessing the need for revaccination is warranted and is an approach currently used for hepatitis B vaccination in patients receiving dialysis [55]; however, such monitoring is not performed for COVID-19 due to poorly defined correlates of protection against the omicron variant [34, 56]. Other potential strategies for enhancing immune protection against COVID-19 after mRNA vaccination include altering the dose level, timing between doses, or providing alternative routes of vaccine administration; however, the efficacy and safety of these approaches have not yet been studied. In addition to inducing immune protection by vaccination, passive immunity with neutralizing monoclonal antibodies has been used as a treatment approach for COVID-19, although the approach is currently not feasible due to the emergence of new SARS-CoV-2 variants [57]. As such, vaccination strategies have progressed to broaden protection against SARS-CoV-2 variants, such as omicron, by developing variant-updated mRNA vaccines against both the ancestral SARS-CoV-2 and a variant of concern (bivalent vaccines) [58]. These vaccines are now recommended as an additional dose in many countries such as the United States [47]. At the time of this writing, no data have been published on the efficacy and safety of these vaccines among patients receiving dialysis. Potential limitations of the data summarized in this review include the retrospective and observational nature of the studies as well as the underrepresentation of pediatric populations and younger adults, as most studies focused on participants >40 years of age.

In conclusion, this review offers a high-level overview of the immunogenicity and effectiveness of mRNA vaccines in patients receiving dialysis that highlights the benefits of vaccination and identifies the unique challenges in achieving and maintaining immune protection in this population. In the midst of an evolving pandemic, routine surveillance and further studies of the impact of vaccination in patients receiving dialysis are warranted to further characterize immune responses to mRNA-based COVID-19 vaccines as well as those using other platforms. Prompt diagnoses and the availability of treatment options will remain important tools to mitigate the high burden of COVID-19 in this population.

## Notes

***Acknowledgments*.** Medical writing and editorial assistance were provided by Agnieszka Looney, PhD, of MEDiSTRAVA in accordance with Good Publication Practice (GPP 2022) guidelines, funded by Moderna, Inc., and under the direction of the authors.

***Supplement sponsorship***. This article appears as part of the supplement “COVID-19 Vaccination in the Immunocompromised Population,” sponsored by Moderna, Inc.

## References

[1] Kovesdy CP . Epidemiology of chronic kidney disease: an update 2022. Kidney Int Suppl (2011) 2022; 12:7–11. 35529086 10.1016/j.kisu.2021.11.003 PMC9073222

[2] Thurlow JS , Joshi M, Yan G, et al Global epidemiology of end-stage kidney disease and disparities in kidney replacement therapy. Am J Nephrol 2021; 52:98–107. 33752206 10.1159/000514550 PMC8057343

[3] Tang H , Tu C, Xiong F, et al Risk factors for the mortality of hemodialysis patients with COVID-19: a multicenter study from the overall hemodialysis population in Wuhan. Semin Dial 2022; 35:71–80. 34137080 10.1111/sdi.12995 PMC8447194

[4] Nguyen DB , Arduino MJ, Patel PR. Hemodialysis-associated infections. In: Himmelfarb J, Ikizler TA, eds. Chronic Kidney Disease, Dialysis, and Transplantation, 4th ed. Philadelphia, PA: Elsevier, 2019:389–410.e8.

[5] El Karoui K , De Vriese AS. COVID-19 in dialysis: clinical impact, immune response, prevention, and treatment. Kidney Int 2022; 101:883–94. 35176326 10.1016/j.kint.2022.01.022 PMC8842412

[6] Altieri P , Sau G, Cao R, et al Immunosuppressive treatment in dialysis patients. Nephrol Dial Transplant 2002; 17(suppl 8):2–9. 10.1093/ndt/17.suppl_8.2 12147770

[7] Saco TV , Strauss AT, Ledford DK. Hepatitis B vaccine nonresponders: possible mechanisms and solutions. Ann Allergy Asthma Immunol 2018; 121:320–7. 29567355 10.1016/j.anai.2018.03.017

[8] Li T , Gandra S, Reske KA, et al Predictors of humoral response to SARS-CoV-2 mRNA vaccine BNT162b2 in patients receiving maintenance dialysis. Antimicrob Steward Healthc Epidemiol 2022; 2:e48. 36310813 10.1017/ash.2022.31 PMC9615013

[9] Van Praet J , Reynders M, De Bacquer D, et al Predictors and dynamics of the humoral and cellular immune response to SARS-CoV-2 mRNA vaccines in hemodialysis patients: a multicenter observational study. J Am Soc Nephrol 2021; 32:3208–20. 34588184 10.1681/ASN.2021070908 PMC8638385

[10] Hsu CM , Weiner DE, Manley HJ, et al Seroresponse to SARS-CoV-2 vaccines among maintenance dialysis patients over 6 months. Clin J Am Soc Nephrol 2022; 17:403–13. 35144972 10.2215/CJN.12250921 PMC8975038

[11] Stumpf J , Schwöbel J, Lindner T, et al Risk of strong antibody decline in dialysis and transplant patients after SARS-CoV-2mRNA vaccination: six months data from the observational Dia-Vacc study. Lancet Reg Health Eur 2022; 17: 100371. 35434688 10.1016/j.lanepe.2022.100371 PMC8995854

[12] Williamson EJ , Walker AJ, Bhaskaran K, et al Factors associated with COVID-19-related death using OpenSAFELY. Nature 2020; 584:430–6. 32640463 10.1038/s41586-020-2521-4 PMC7611074

[13] Semenzato L , Botton J, Drouin J, et al Characteristics associated with the residual risk of severe COVID-19 after a complete vaccination schedule: a cohort study of 28 million people in France. Lancet Reg Health Eur 2022; 19:100441. 35789881 10.1016/j.lanepe.2022.100441 PMC9243470

[14] Weiss A , Hendrickx R, Stensgaard E, Jellingsø M, Sommer MOA. Kidney transplant and dialysis patients remain at increased risk for succumbing to COVID-19. Transplantation 2023; 107:1136–8. 36584380 10.1097/TP.0000000000004462 PMC10125010

[15] Peiyao R , Mengjie Y, Xiaogang S, et al Immunogenicity and safety of SARS-CoV-2 vaccine in hemodialysis patients: a systematic review and meta-analysis. Front Public Health 2022; 10:951096. 36211647 10.3389/fpubh.2022.951096 PMC9539993

[16] Falahi S , Sayyadi H, Kenarkoohi A. Immunogenicity of COVID-19 mRNA vaccines in hemodialysis patients: systematic review and meta-analysis. Health Sci Rep 2022; 5:e874. 36210877 10.1002/hsr2.874 PMC9528953

[17] Ma BM , Tam AR, Chan KW, et al Immunogenicity and safety of COVID-19 vaccines in patients receiving renal replacement therapy: a systematic review and meta-analysis. Front Med (Lausanne) 2022; 9:827859. 35355604 10.3389/fmed.2022.827859 PMC8959490

[18] Polack FP , Thomas SJ, Kitchin N, et al Safety and efficacy of the BNT162b2 mRNA Covid-19 vaccine. N Engl J Med 2020; 383:2603–15. 33301246 10.1056/NEJMoa2034577 PMC7745181

[19] Baden LR , El Sahly HM, Essink B, et al Efficacy and safety of the mRNA-1273 SARS-CoV-2 vaccine. N Engl J Med 2021; 384:403–16. 33378609 10.1056/NEJMoa2035389 PMC7787219

[20] Sanders JF , Bemelman FJ, Messchendorp AL, et al The RECOVAC immune-response study: the immunogenicity, tolerability, and safety of COVID-19 vaccination in patients with chronic kidney disease, on dialysis, or living with a kidney transplant. Transplantation 2022; 106:821–34. 34753894 10.1097/TP.0000000000003983 PMC8942603

[21] Garcia P , Anand S, Han J, et al COVID-19 vaccine type and humoral immune response in patients receiving dialysis. J Am Soc Nephrol 2022; 33:33–7. 34645698 10.1681/ASN.2021070936 PMC8763174

[22] Dimitrov Y , Krummel T, Chantrel F, et al Protective antibody response to mRNA-1273 and BNT162b2 vaccines in patients on maintenance haemodialysis: a prospective cohort study. Clin Kidney J 2022; 15:1720–6. 35999964 10.1093/ckj/sfac082 PMC9383598

[23] Anand S , Montez-Rath ME, Han J, et al Antibody response to COVID-19 vaccination in patients receiving dialysis. J Am Soc Nephrol 2021; 32:2435–8. 34117129 10.1681/ASN.2021050611 PMC8722791

[24] Hsu CM , Weiner DE, Aweh GN, et al Seroresponse to SARS-CoV-2 vaccines among maintenance dialysis patients. Am J Kidney Dis 2022; 79:307–10. 34758369 10.1053/j.ajkd.2021.10.002 PMC8572553

[25] Anand S , Montez-Rath ME, Han J, et al SARS-CoV-2 vaccine antibody response and breakthrough infection in patients receiving dialysis. Ann Intern Med 2022; 175:371–8. 34904856 10.7326/M21-4176 PMC8722718

[26] Grupper A , Sharon N, Finn T, et al Humoral response to the Pfizer BNT162b2 vaccine in patients undergoing maintenance hemodialysis. Clin J Am Soc Nephrol 2021; 16:1037–42. 33824157 10.2215/CJN.03500321 PMC8425628

[27] Speer C , Goth D, Benning L, et al Early humoral responses of hemodialysis patients after COVID-19 vaccination with BNT162b2. Clin J Am Soc Nephrol 2021; 16:1073–82. 34031181 10.2215/CJN.03700321 PMC8425619

[28] Bassi J , Giannini O, Silacci-Fregni C, et al Poor neutralization and rapid decay of antibodies to SARS-CoV-2 variants in vaccinated dialysis patients. PLoS One 2022; 17:e0263328. 35143540 10.1371/journal.pone.0263328 PMC8830698

[29] Clavero R , Parra-Lucares A, Méndez-Valdés G, et al Humoral immune response of BNT162b2 and CoronaVac vaccinations in hemodialysis patients: a multicenter prospective cohort. Vaccines (Basel) 2022; 10:1542. 36146621 10.3390/vaccines10091542 PMC9503801

[30] Raja N , Rajagopalan A, Arunachalam J, Prasath A, Durai R, Rajendran M. Humoral response to viral vector COVID-19 vaccine in hemodialysis patients. Kidney Res Clin Pract 2022; 41:342–50. 35286797 10.23876/j.krcp.21.184 PMC9184837

[31] Pollard AJ , Bijker EM. A guide to vaccinology: from basic principles to new developments. Nat Rev Immunol 2021; 21:83–100. 33353987 10.1038/s41577-020-00479-7 PMC7754704

[32] Stumpf J , Siepmann T, Lindner T, et al Humoral and cellular immunity to SARS-CoV-2 vaccination in renal transplant versus dialysis patients: a prospective, multicenter observational study using mRNA-1273 or BNT162b2 mRNA vaccine. Lancet Reg Health Eur 2021; 9:100178. 34318288 10.1016/j.lanepe.2021.100178 PMC8299287

[33] Piotrowska M , Zieliński M, Tylicki L, et al Local and systemic immunity are impaired in end-stage-renal-disease patients treated with hemodialysis, peritoneal dialysis and kidney transplant recipients immunized with BNT162b2 Pfizer-BioNTech SARS-CoV-2 vaccine. Frontiers in Immunology 2022; 13:832924. 35935974 10.3389/fimmu.2022.832924 PMC9354587

[34] Centers for Disease Control and Prevention . COVID-19 vaccines for moderately or severely immunocompromised people. https://www.cdc.gov/coronavirus/2019-ncov/vaccines/recommendations/immuno.html. Accessed 21 April 2022.

[35] World Health Organization . Interim recommendations for an extended primary series with an additional vaccine dose for COVID-19 vaccination in immunocompromised persons. https://www.who.int/publications/i/item/WHO-2019-nCoV-vaccines-SAGE_recommendation-immunocompromised-persons. Accessed 1 March 2023.

[36] World Health Organization . Interim statement on the use of additional booster doses of emergency use listed mRNA vaccines against COVID-19. https://www.who.int/news/item/17-05-2022-interim-statement-on-the-use-of-additional-booster-doses-of-emergency-use-listed-mrna-vaccines-against-covid-19. Accessed 1 March 2023.

[37] Kohmer N , Rabenau HF, Ciesek S, et al Heterologous immunization with BNT162b2 followed by mRNA-1273 in dialysis patients: seroconversion and presence of neutralizing antibodies. Nephrol Dial Transplant 2022; 37:1132–9. 35099023 10.1093/ndt/gfac018 PMC9383412

[38] Beilhack G , Monteforte R, Frommlet F, Reindl-Schwaighofer R, Strassl R, Vychytil A. Humoral response to mRNA-1273 SARS-CoV-2 vaccine in peritoneal dialysis patients: is boostering after six months adequate? Front Med (Lausanne) 2022; 9:905798. 35814775 10.3389/fmed.2022.905798 PMC9263093

[39] Patyna S , Eckes T, Koch BF, et al Impact of Moderna mRNA-1273 booster vaccine on fully vaccinated high-risk chronic dialysis patients after loss of humoral response. Vaccines (Basel) 2022; 10:585. 35455334 10.3390/vaccines10040585 PMC9029590

[40] Wang X , Han M, Fuentes LR, et al SARS-CoV-2 neutralizing antibody response after three doses of mRNA1273 vaccine and COVID-19 in hemodialysis patients. Front Nephrol. 2022; 2:926635. 36106337 10.3389/fneph.2022.926635 PMC9470295

[41] Quiroga B , Soler MJ, Ortiz A, et al Anti-spike antibodies 3 months after SARS-CoV-2 mRNA vaccine booster dose in patients on hemodialysis: the prospective SENCOVAC study. Clin Kidney J 2022; 15:1856–64. 36147708 10.1093/ckj/sfac169 PMC9384616

[42] Garcia P , Han J, Montez-Rath ME, et al SARS-CoV-2 booster vaccine response among patients receiving dialysis. Clin J Am Soc Nephrol 2022; 17:1036–8. 35383042 10.2215/CJN.00890122 PMC9269633

[43] Becker M , Cossmann A, Lurken K, et al Longitudinal cellular and humoral immune responses after triple BNT162b2 and fourth full-dose mRNA-1273 vaccination in haemodialysis patients. Front Immunol 2022; 13:1004045. 36275672 10.3389/fimmu.2022.1004045 PMC9582343

[44] Affeldt P , Koehler FC, Brensing KA, et al Immune response to third and fourth COVID-19 vaccination in hemodialysis patients and kidney transplant recipients. Viruses 2022; 14:2646. 36560648 10.3390/v14122646 PMC9785871

[45] Broseta JJ , Rodriguez-Espinosa D, Cuadrado E, Rodriguez N, Bedini JL, Maduell F. Humoral response after three doses of mRNA-1273 or BNT162b2 SARS-CoV-2 vaccines in hemodialysis patients. Vaccines (Basel) 2022; 10:585. 35455271 10.3390/vaccines10040522 PMC9030003

[46] Quiroga B , Soler MJ, Ortiz A, et al Humoral response after the fourth dose of the SARS-CoV-2 vaccine in the CKD spectrum: a prespecified analysis of the SENCOVAC study. Nephrol Dial Transplant 2023; 38:969–81. 36423334 10.1093/ndt/gfac307

[47] Rosenblum HG , Wallace M, Godfrey M, et al Interim recommendations from the advisory committee on immunization practices for the use of bivalent booster doses of COVID-19 vaccines - United States, October 2022. MMWR Morb Mortal Wkly Rep 2022; 71:1436–41. 36355612 10.15585/mmwr.mm7145a2 PMC9707353

[48] Huth L , Schäfer L, Almanzar G, et al Immunologic effect of bivalent mRNA booster in patients undergoing hemodialysis. N Engl J Med 2023; 388:950–2. 36791308 10.1056/NEJMc2216309 PMC9944762

[49] Sibbel S , McKeon K, Luo J, et al Real-world effectiveness and immunogenicity of BNT162b2 and mRNA-1273 SARS-CoV-2 vaccines in patients on hemodialysis. J Am Soc Nephrol 2022; 33:49–57. 34789546 10.1681/ASN.2021060778 PMC8763185

[50] Manley HJ , Li NC, Aweh GN, et al SARS-CoV-2 vaccine effectiveness and breakthrough infections among patients receiving maintenance dialysis. Am J Kidney Dis 2022; 81:406–15. 36462570 10.1053/j.ajkd.2022.10.010 PMC9711902

[51] Mosconi G , Fantini M, Righini M, et al Efficacy of SARS-CoV-2 vaccination in dialysis patients: epidemiological analysis and evaluation of the clinical progress. J Clin Med 2022; 11:4723. 36012962 10.3390/jcm11164723 PMC9410204

[52] Ashby DR , Caplin B, Corbett RW, et al Severity of COVID-19 after vaccination among hemodialysis patients: an observational cohort study. Clin J Am Soc Nephrol 2022; 17:843–50. 35649718 10.2215/CJN.16621221 PMC9269655

[53] Ichii M , Kurajoh M, Okute Y, et al Reduced risk of progression from non-severe to severe COVID-19 in hospitalized dialysis patients by full COVID-19 vaccination. J Clin Med 2022; 11:6348. 36362579 10.3390/jcm11216348 PMC9657170

[54] Yang X , Zhang H, Bao W, Fu S, Jin H. Immunogenicity rates after SARS-CoV-2 three-dose vaccination in patients under dialysis: a systematic review and meta-analysis. Vaccines (Basel) 2022; 10:2070. 36560480 10.3390/vaccines10122070 PMC9782384

[55] Centers for Disease Control and Prevention . Guidelines for vaccinating dialysis patients and patients with chronic kidney disease; summarized from recommendations of the Advisory Committee on Immunization Practices (ACIP). https://stacks.cdc.gov/view/cdc/13599. Accessed 8 March 2023.

[56] Khoury D , Schlub T, Cromer D, et al Correlates of protection, thresholds of protection, and immunobridging among persons with SARS-CoV-2 infection. Emerg Infect Dis 2023; 29:381–8. 36692375 10.3201/eid2902.221422 PMC9881762

[57] Yamasoba D , Kosugi Y, Kimura I, et al Neutralisation sensitivity of SARS-CoV-2 omicron subvariants to therapeutic monoclonal antibodies. Lancet Infect Dis 2022; 22:942–3. 35690075 10.1016/S1473-3099(22)00365-6 PMC9179126

[58] Davis-Gardner ME , Lai L, Wali B, et al Neutralization against BA.2.75.2, BQ.1.1, and XBB from mRNA bivalent booster. N Engl J Med 2023; 388:183–5. 36546661 10.1056/NEJMc2214293 PMC9812288

